# ﻿Contribution to the knowledge of the longicorn beetle genus *Neocerambyx* (Coleoptera, Cerambycidae, Cerambycinae, Cerambycini) from China, with descriptions of three new species

**DOI:** 10.3897/zookeys.1247.153858

**Published:** 2025-07-22

**Authors:** Mei-Ying Lin, Alexandr Miroshnikov, Li He

**Affiliations:** 1 Engineering Research Center for Forest and Grassland Disaster Prevention and Reduction / Ecological Security and Protection Key Laboratory of Sichuan Province, school of Life Sciences (school of Ecological Forestry), Mianyang Normal University Mianyang Normal University Mianyang China; 2 Russian Entomological Society, Krasnodar, Russia Russian Entomological Society Krasnodar Russia; 3 State Grid Tianfu New Area Electric Power Supply Company, Chengdu, Sichuan 610094, China State Grid Tianfu New Area Electric Power Supply Company Chengdu China

**Keywords:** Cerambycidae, China, *Neocerambyx*, new records, new species, taxonomy

## Abstract

The genus *Neocerambyx* J. Thomson, 1861 in China is surveyed, and three new species are described: *N.liyuani* Lin, Miroshnikov & He, **sp. nov.** from Sichuan and Hubei provinces, *N.miaobenfui* Lin, Miroshnikov & He, **sp. nov.** and *N.gui* Lin, Miroshnikov & He, **sp. nov.**, both from Hainan Island. *Neocerambyxmelas* (Holzschuh, 2021) is recorded from China (Fujian province) for the first time.

## ﻿Introduction

The genus *Neocerambyx* J. Thomson, 1861 has been studied and discussed quite frequently in the last years (Lazarev 2019, 2020; Miroshnikov 2020a, b, 2021b, 2022), with several new species being described (Holzschuh 2020; Jacquot 2020; Li et al. 2020; Miroshnikov 2021a). It currently includes 24 valid species and two subspecies (Tavakilian and Chevillotte 2024). The genus can be divided into six species groups (Miroshnikov 2020b):

1. The *paris* group (7 described and 1 new species): *N.paris* (Wiedemann, 1821), *N.gigas* (J. Thomson, 1878), *N.grandis* Gahan, 1891, *N.luzonicus* Hüdepohl, 1987, *N.opulentus* Holzschuh, 1998, *N.katarinae* Holzschuh, 2009, *N.paulae* Miroshnikov, 2021a, and *N.liyuani* sp. nov.
2. The *unicolor* group (6 described and 2 new species): *N.unicolor* (Gahan, 1906), *N.vitalisi* Pic, 1923, *N.elenae* Lazarev, 2019, *N.brudermanni* Holzschuh, 2020, *N.punctulifer* Holzschuh, 2020, *N.sabinae* Holzschuh, 2020, *N.miaobenfui* sp. nov., and *N.gui* sp. nov.
3. The *pellitus* group (6 described species): *N.pellitus* (Hitzinger, 1943), *N.theresae* (Pic, 1946), *N.rugicollis* Gressitt, 1948, *N.bakboensis* Miroshnikov, 2018, *N.zubrzyckii* Miroshnikov, 2021a, and *N.melas* (Holzschuh, 2021).
4. The *dierli* group (3 described species): *N.dierli* (Heyrovský, 1976), *N.atratulus* (Holzschuh, 2018), and *N.gracilipes* Jacquot, 2020.
5. The *pubescens* group: *N.pubescens* Fisher, 1936.
6. The *raddei* group: *N.radddei* Blessig, 1872.

Eleven species / subspecies of *Neocerambyx* have been recorded from China (Chen et al. 2019; Lazarev 2019; Jacquot 2020; Li et al. 2020; Miroshnikov 2020a). In the present paper, three new species are described from China and one species is newly recorded from China, which raises the species number to 15.

## ﻿Materials and methods

Habitus images of *Neocerambyxliyuani* sp. nov., *N.gigas* and *N.miaobenfui* sp. nov. were taken using a Canon 50D DSLR with a Canon EF 100 mm f/2.8L IS USM lens, and a dual LED fill light was used as the light source. Images of the same object at different focal planes were combined using Zerene Stacker v. 1.04 stacking software. Other habitus photographs were taken with a Canon EOS 7D camera with a Canon Macro 100 mm lens. Images of the same object at different focal planes were combined using Helicon Focus v. 8 stacking software. Adobe Photoshop CS6 was used for postprocessing.

Specimens studied are deposited in the following institutional and private collections:

**CAM** collection of Alexandr Miroshnikov, Krasnodar, Russia;

**CBWX** collection of Wen-Xuan Bi, Shanghai, China;

**CCCC** collection of Chang-Chin Chen, Tianjin, China;

**CLHC** collection of Li He, Chengdu, Sichuan, China;

**CLYQ** Chonglinyequ, Fuzhou, Fujian, China;

**CWD** collection of Dong Wen, Qingdao, Shandong, China;

**CYLD** Collection of Yuan Li, Deyang, Sichuan, China;

**IZCAS**Institute of Zoology, Chinese Academy of Sciences [= NACRC National Animal Collection Resource Center], Beijing, China;

**MYNU** Invertebrate collection of Mianyang Normal University, Mianyang, Sichuan, China.

## ﻿Taxonomic account

### ﻿Genus Neocerambyx

Taxon classificationAnimaliaColeopteraCerambycidae

J. Thomson, 1861

F3AF9242-2388-519F-8F4E-E72EE3CCFD09

Neocerambyx J. Thomson, 1861: 194. Type species: Cerambyxparis Wiedemann, 1821, designated by J. Thomson (1864: 231).
Mallambyx (subgenus of Pachydissus) Bates, 1873: 152. Type species: Pachydissus (Mallambyx) japonicus Bates, 1873 (= Neocerambyxraddei Blessig, 1872), by monotypy. Synonymized by Hüdepohl (1990: 249) not by Gressitt and Rondon (1970: 59), who synonymized Mallambyx with Massicus.
Mesocerambyx Hitzinger in Breuning and Hitzinger 1943: 37. Type species: Mesocerambyxpellitus Hitzinger in Breuning and Hitzinger 1943, by original designation. Synonymized by Hüdepohl (1990: 248).
Falsomassicus Pic, 1946: 7. Type species: Falsomassicustheresae Pic, 1946, by monotypy. Synonymized by Miroshnikov (2020a: 79) not by Gressitt and Rondon (1970: 59), who synonymized Falsomassicus with Massicus. Synonymy discussed by Miroshnikov (2021a: 291, 2021b: 482).
Bulbocerambyx Lazarev, 2019: 1194. Type species: Neocerambyxgrandis Gahan, 1891, by original designation. Synonymized by Miroshnikov (2020a: 81). Reinstated by Lazarev (2020: 123). Synonymy confirmed/accepted by Miroshnikov (2020b: 377, 2021b: 469), Li et al. (2020: 582), Vitali (2022: 3), and Tavakilian and Chevillotte (2024).

#### Distribution.

Oriental Region and the East Asian (or Himalayan-Chinese) subregion of the Palaearctic Region, including the south of the Russian Far East.

#### Remarks.

We follow Miroshnikov (2020a, b) for the definition of the genus, and we treat *Bulbocerambyx* Lazarev, 2019 as its junior synonym.

### ﻿ Neocerambyx liyuani

Taxon classificationAnimaliaColeopteraCerambycidae

Lin, Miroshnikov & He sp. nov.

54525AB8-5636-5547-9E02-9456373E7958

https://zoobank.org/F0411080-989F-4038-911D-2692CE6F0B19

Figs 1A–D , 2A, B

#### Type material.

***Holotype***: China • ♂; Sichuan, Liangshan Yi Autonomous Prefecture, Leibo County, Guihua Village [四川省凉山彝族自治州雷波县桂花乡]; alt. 1740 m; 15 May 2024; Yuan Li leg.; at light; MYNU. ***Paratypes***: China • 1♀, same data as for holotype; 16 May 2024; MYNU • 1♂, 1♀; same data as for holotype; 24 May 2024; CAM • 1♂, 1♀; same data as for holotype; 19 May 2025; Chun-Nan Li leg.; CLHC • 1♂; same data as for holotype; 20 May 2025; Chun-Nan Li leg.; CYLD • 1♀; Hubei, Shennongjia, Muyuzhen [湖北神农架木鱼镇]; 4 July 1993; IZCAS.

#### Diagnosis.

**Figure 1.**
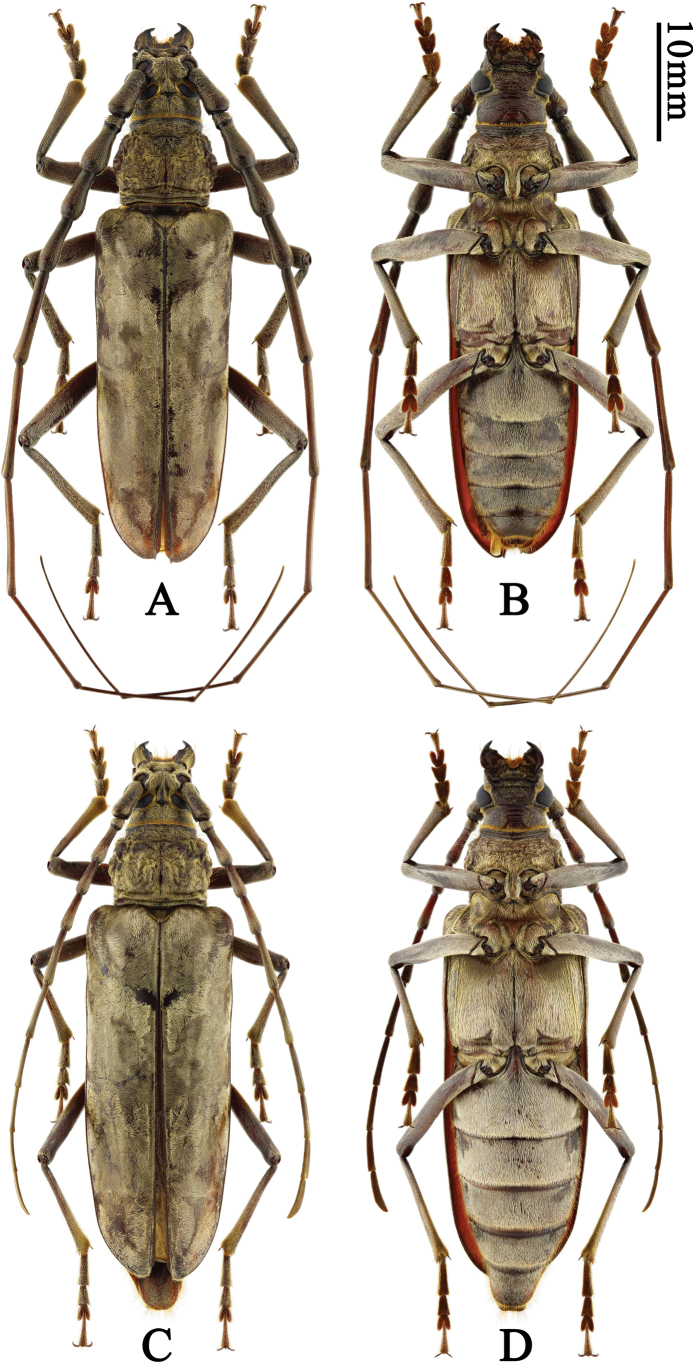
Habitus of *Neocerambyxliyuani* sp. nov. **A, B.** ♂, holotype, from Sichuan; **C, D.** ♀, paratype, from Sichuan: **A, C.** Dorsal views; **B, D.** Ventral views.

This new species is rather peculiar by the shining pubescence and particularly the male somewhat resembles a miniature *Neocerambyxgigas* (Fig. 3A–D), but differs from that species by the presence of a short transverse groove between upper eye lobes; the smaller body size (*N.gigas* measures 55–84 mm in length); the features of the sculpture of the pronotal disc; the more slender elytra of both sexes (cf. Figs 1A and 3A); the longer female antennae (cf. Figs 1C and 3C), and the shape of the external apical angle of female antennomeres 8–10 and of the apex of female last antennomere (in *N.gigas*, the external apical angle of female antennomeres 8–10 is very sharp and the distal part of female last antennomere sharply tapers towards the apex).

The new species should be attributed to the *paris* group sensu Miroshnikov (2020b): the anterior coxal cavities externally with a large triangular protrusion, the external apical angle of antennomeres 5–10 without a sharp spine, the elytra with a recumbent setation forming an iridescent pattern.

#### Description.

Body length 43.0–47.0 mm, humeral width 12.0–12.5 mm. Body black to black-brown, covered with a golden yellow shining pubescence. Head with pubescence especially dense around compound eyes.

All antennomeres black-brown, without annulations, covered with golden yellow pubescence, without a fringe of setae underneath. Male antennae exceed apex of elytra by approximately the middle of antennomere 8; scape stout, subequal to third antennomere in length; third to fifth antennomeres distinctly inflated in apical part (inflation decreases from third to fifth); third subequal to fourth in length, sixth and seventh antennomeres slender and cylindrical, sixth much longer than fifth, slightly shorter than fourth and fifth combined, seventh longer than sixth, subequal to fourth and fifth combined; from eighth to eleventh, antennomeres become flatter and more slender, eighth to tenth subequal in length, eleventh the longest, much longer than tenth. Female antennae distinctly fail to reach apex of elytra; scape stout, subequal to third antennomere in length, third to fifth antennomeres slightly expanded in apical part, third longer than fourth; fifth slightly longer than fourth, and slightly shorter than third; sixth and seventh antennomeres slender and cylindrical, sixth obviously longer than fifth and obviously shorter than fourth and fifth combined, seventh slightly shorter and more slender than sixth; from eighth to eleventh, antennomeres become flatter but not more slender, eighth shorter than seventh, ninth shorter than eighth, tenth slightly shorter than ninth, eleventh and tenth subequal in length; sixth is the longest antennomere.

Eye deeply emarginate, lower lobe very large; head with a short transverse groove between upper eye lobes. Mandible moderately sized, curved and sharp apically, with one blunt mesal tooth. Prothorax covered with dense golden yellow pubescence, fringed with short orange setae at anterior and posterior margins of pronotum, and with a few long, erect setae scattered on sides. Pronotum 1.26 and 1.24× as wide as long in male and female, respectively; at base distinctly wider than at apex; usually with an abrupt constriction at apex and a moderate constriction at base; pronotum with coarse, irregular (but in some regions largely transverse) grooves, with a smooth medial area in basal half.

Scutellum covered with golden yellow pubescence, with rounded triangular posterior angle. Elytra completely covered with golden recumbent pubescence forming an iridescent pattern; moderately elongate, 2.60–2.64× as long as humeral width; approximately parallel-sided from base, rounded at apex. Venter with pubescence scattered over most sclerites. Prosternum with a deep transverse groove in front of middle, prosternal intercoxal process with a distinct apical tubercle, particularly distinct in males. Mesoventral intercoxal process with pubescence denser on sides than middle part; between coxae very clearly wider than prosternal process; metasternum with a very sharp longitudinal median groove. Legs black-brown, moderately long; femora and tibiae quite robust in male; metatarsomere 1 barely longer than tarsomeres 2 and 3 combined. Last visible abdominal sternite at apex in male with a shallow emargination, in female widely rounded; last visible abdominal tergite at apex in male narrowly and shallowly emarginate, in female widely rounded.

#### Etymology.

The specific epithet is gratefully dedicated to the collector of the type specimens from Sichuan, Mr Yuan Li (李圆, Deyang, Sichuan, China), an enthusiastic amateur entomologist and an experienced collector of beetles. The name is a noun in the genitive case.

#### Distribution.

China: Sichuan, Hubei.

#### Biology.

**Figure 2.**
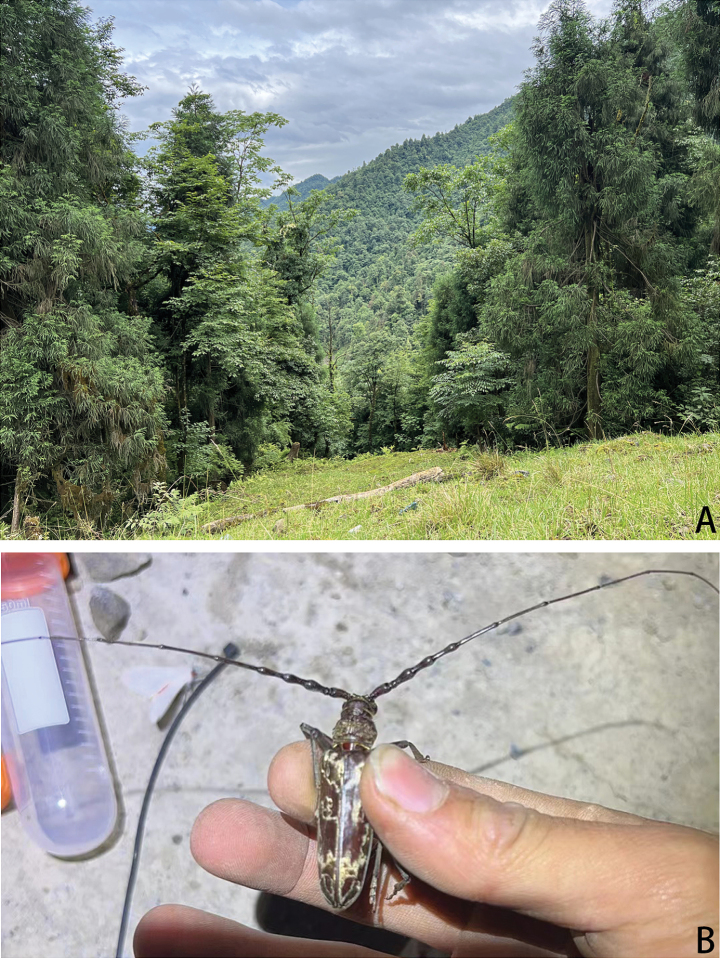
Field observations of *Neocerambyxliyuani* sp. nov. **A.** Habitat of Guihua Village (Sichuan, CHINA); **B.** A living male caught by Yuan Li (all provided by Yuan Li).

Seven specimens were collected by a light trap (Fig. 2B) installed in the forest environment (Fig. 2A).

**Figure 3.**
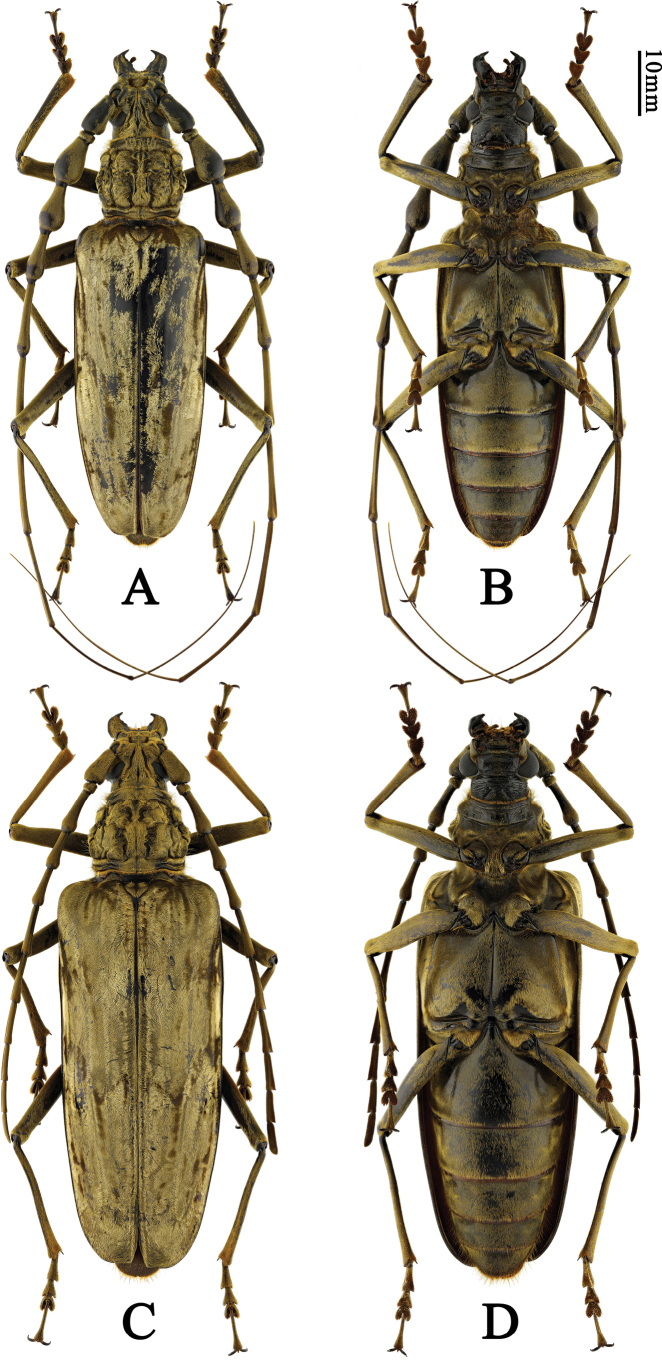
Habitus of *Neocerambyxgigas* (Thomson, 1878). **A, B.** ♂, from Chiang Mai, Thailand; **C, D.** ♀, from Chiang Mai, Thailand: **A, C.** Dorsal views; **B, D.** Ventral views.

### ﻿ Neocerambyx miaobenfui

Taxon classificationAnimaliaColeopteraCerambycidae

Lin, Miroshnikov & He sp. nov.

5BB840B8-7C75-57A9-AF3D-E1F65DD35607

https://zoobank.org/42F698B7-9F65-4679-9395-F74EA26C4F9B

Fig. 4A–D

#### Type material.

***Holotype***: China • ♂; Hainan, Ledong Li Autonomous County, Jianfeng Town, Jianfengling National Forest Park [海南省乐东黎族自治县尖峰镇尖峰岭国家森林公园]; alt. 940 m; 18°44'38″N, 108°50'38″E; 20 April-10 May 2024; Sheng Wu leg.; at light; MYNU. ***Paratypes***: China • 1♂; same data as for holotype; CAM • 1♂, 1♀; Hainan, Ledong County, Jianfengling, Tianchi Bishushanzhuang [海南省乐东县尖峰岭天池避暑山庄]; alt. 950 m; 26 May 2011; Wenhsin Lin leg.; IZCAS • 1♂, 1♀; same data as for preceding; 21–22 May 2011 • 1♂, 2♀♀; same data as for preceding; 29 May 2011; CCCC • 1♀; Ledong, Jianfengling, Tianchi [尖峰岭天池]; alt. 808 m; Ke-Qing Song leg.; by light trap; IZCAS; IOZ(E)1883507) • 1♂; Ledong, Jianfengling, Tianchi [尖峰岭天池]; alt. 800 m; May 2017; local collector leg.; CLHC • 1♂; Ledong County, Jianfengling Nat. Rev., Mingfenggu [海南省乐东县尖峰岭自然保护区鸣凤谷]; alt. 950 m; 09 June 2017; local collector leg.; CWD • 1 ♀; Ledong, Jianfengling, Mingfenggu [海南省乐东县尖峰岭鸣凤谷]; alt. 950–1,000 m; 22 May 2011; Wen-Xuan Bi leg.; CBWX • 1♀; Jianfengling, Yulingu [尖峰岭雨林谷]; alt. 746 m; 18°45'20″N, 108°53'50″E; 19 May 2009; Ke-Qing Song leg.; IZCAS; IOZ(E)1883508.

**Figure 4.**
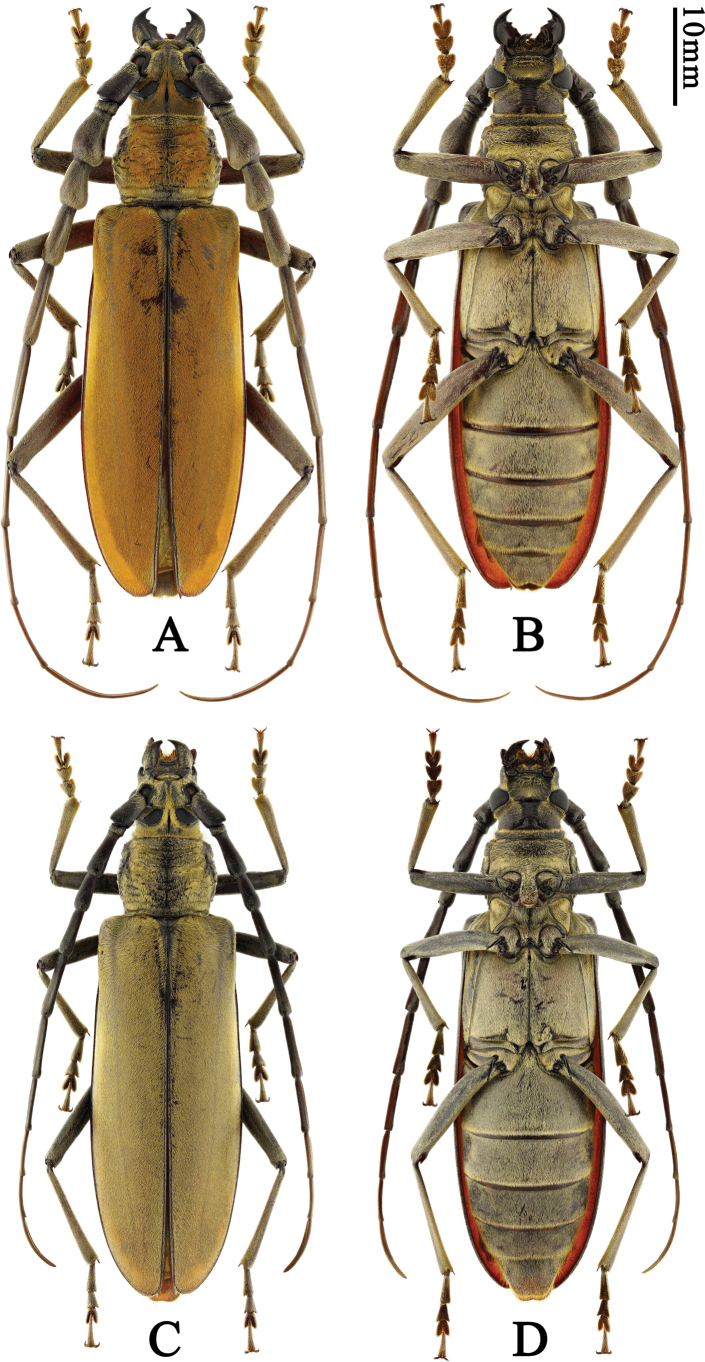
Habitus of *Neocerambyxmiaobenfui* sp. nov. **A, B.** ♂, holotype, from Hainan; **C, D.** ♀, paratype, from Hainan: **A, C.** Dorsal views; **B, D.** Ventral views.

#### Diagnosis.

This new species, in combination with the structure of male antennomeres 3 and 4, the sculpture of the pronotal disc, and the setation of the elytra, somewhat resembles *Neocerambyxsabinae*, but differs by the less strongly protruding external apical angle of antennomere 1, the scutellum being more sharply narrowed towards the apex, the silver-grey recumbent setation of the antennae and legs, poorly hiding a black or dark coloration of the cuticle (in *N.sabinae*, the recumbent setation of the antennae and legs is of yellow tones and strongly or significantly hiding the black or dark coloration of the cuticle), the less strongly developed antennal tubercles, the shorter male antennae (and thus less elongate antennomeres), and the smaller body size (*N.sabinae* measures 73–79 mm in length).

*Neocerambyxmiaobenfui* sp. nov. can also be compared to *N.unicolor* (Gahan, 1906), *N.vitalisi* Pic, 1923, *N.punctulifer* Holzschuh, 2020, and *N.brudermanni* Holzschuh, 2020. Like in *N.sabinae*, it can be distinguished by the less strongly protruding external apical angle of antennomere 1 and the shorter male antennae (and thus less elongate antennomeres, including 3–5). In addition, *N.miaobenfui* differs from *N.unicolor* and *N.vitalisi* by the more strongly inflated apical part of male antennomere 4, and from *N.punctulifer* and *N.brudermanni* by the more strongly inflated apical parts of male antennomeres 3 and 4.

The new species should be attributed to the *unicolor* group sensu Miroshnikov (2020b), which includes all the species discussed above. The anterior coxal cavities are externally with a large triangular protrusion and the external apical angle of antennomeres 5–10 is without a sharp spine, which is the same as the *paris* group, but “elytra with a uniform recumbent setation that does not form an iridescent pattern” (Miroshnikov 2020b).

#### Description.

Body length 47.5–64.0 mm, humeral width 14.0–18.0 mm. Body black to black-brown or dark brown, covered with orange yellow to ochraceous pubescence. Head with pubescence, especially denser around eyes.

Most of antennomeres black starting from scape, last few antennomeres somewhat lightened, all antennomeres without annulations, covered with ochraceous or yellow pubescence, without a fringe of setae underneath. Male antennae exceed apex of elytra by antennomere 9; scape stout, barely shorter than third antennomere; third and fourth antennomeres (third in particular) strongly and asymmetrically inflated in apical part, without irregular rugose sculpture; fifth distinctly more slender than fourth but stouter than sixth, and distinctly longer than fourth whereas shorter than sixth; sixth to eleventh antennomeres become gradually more slender and flatter, seventh longer than sixth, shorter than fourth and fifth combined; eighth and ninth antennomeres subequal in length, eleventh is the longest, much longer than tenth. Female antennae slightly fail to reach the apex of elytra; scape stout, subequal to fourth antennomere in length; third to fifth antennomeres slightly expanded apically, third and fifth subequal and both longer than fourth; sixth to eleventh antennomeres become gradually more slender and flatter, sixth distinctly longer than fifth, distinctly shorter than fourth and fifth combined, seventh subequal to sixth in length; eighth shorter than seventh, ninth slightly shorter than eighth, tenth slightly shorter than ninth, eleventh is the longest antennomere.

Eye deeply emarginate, lower lobe very large; head with a short longitudinal groove between upper eye lobes and on occiput. Mandible moderately sized, curved and sharp apically, with two mesal teeth. Prothorax covered with dense orange yellow or ochraceous pubescence, fringed with short ochraceous setae at anterior margin of pronotum and a few long, erect setae scattered on sides. Pronotum 1.29 or 1.28× as wide as long in male and female, respectively; at base slightly wider than at apex; usually with an abrupt constriction at apex and a moderate constriction at base; pronotum with coarse, irregular (in some regions largely transverse) grooves, usually ~10 in number.

Scutellum covered with ochraceous pubescence, posterior angle roundly triangular. Elytra moderately elongate, 2.70–2.82× as long as humeral width; approximately parallel-sided from base, apex rounded, apical external angle obtuse, sutural angle with a short tooth. Venter with most sclerites pubescent. Prosternal intercoxal process with a pair of blunt tubercles (more distinct in males), expanded posteriorly; mesoventral intercoxal process entirely covered with pubescence, emarginate and forming two lobes apically, between coxae distinctly wider than prosternal process; metasternum with a very thin median groove. Legs black, partly black-brown, moderately long; femora quite robust, tibiae and tarsi slender; metafemur reaching middle of third visible abdominal sternite; metatarsomere 1 shorter than tarsomeres 2 and 3 combined. Both sexes with last visible abdominal sternite at apex with a shallow emargination; last visible tergite emarginate apically.

#### Etymology.

The specific epithet is gratefully dedicated to the third author’s friend, Mr Ben-Fu Miao (缪本福, Fuzhou, China), an enthusiastic amateur entomologist, who constantly assisted us with his research. The name is a noun in the genitive case.

#### Distribution.

China: Hainan.

### ﻿ Neocerambyx gui

Taxon classificationAnimaliaColeopteraCerambycidae

Lin, Miroshnikov & He sp. nov.

F0D3BF10-3AD4-5E34-87F9-7F8A88381FB1

https://zoobank.org/514BE67C-2705-4F2F-9D4D-5693DC223857

Fig. 5A–D

#### Type material.

***Holotype***: : China • ♂; Hainan, Limushan [海南黎母山]; 28 May 1984; Mao-Bin Gu leg.; by light trap; IZCAS. ***Paratypes***: China • 1♀; same data as for holotype; • 1♀; Hainan, Jianfenglingding [海南尖峰岭顶]; 27 May 1983; Mao-Bin Gu leg.; IZCAS • 1♀; Hainan, Wuzhishan [海南五指山]; alt. 800 m; 2 June 1997; Pei-Yu Yu leg.; IZCAS.

**Figure 5.**
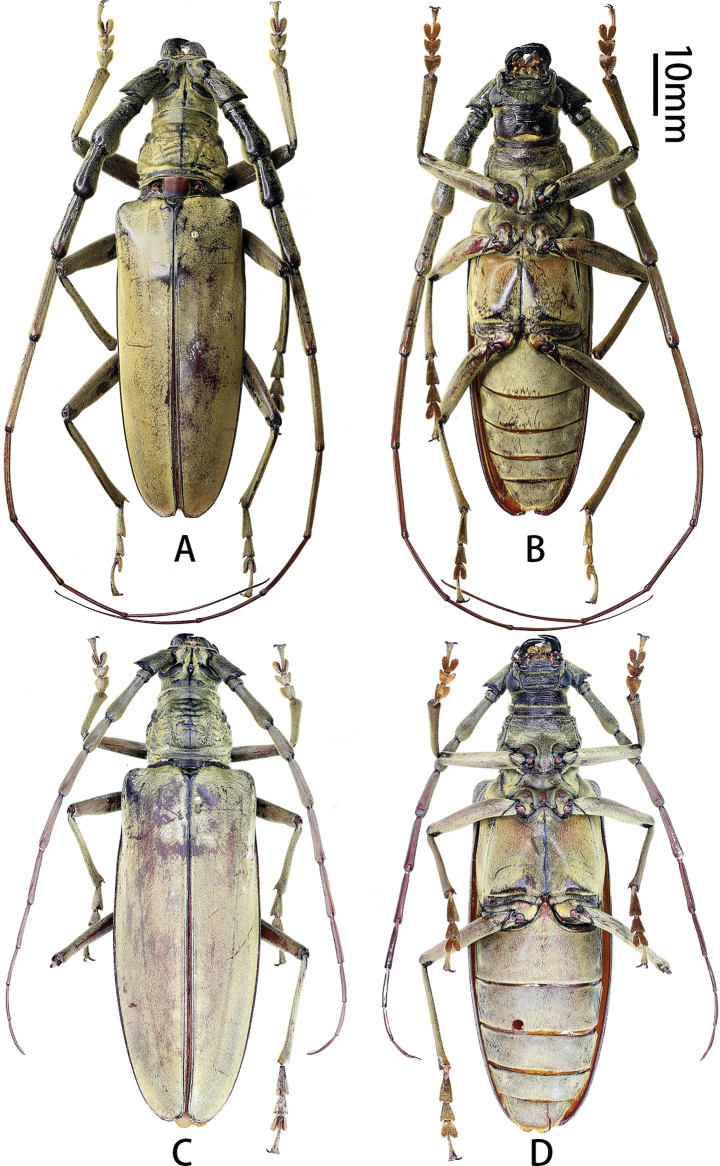
Habitus of *Neocerambyxgui* sp. nov. **A, B.** ♂, holotype, from Hainan; **C, D.** ♀, paratype, from Hainan: **A, C.** Dorsal views; **B, D.** Ventral views.

#### Diagnosis.

The new species is especially similar to *Neocerambyxpunctulifer* Holzschuh, 2020, but differs by the sculpture of the pronotal disc, namely, the more strongly expressed median groove, two oblique incomplete grooves on either side, and the sparser and longer transverse folds and grooves; it further differs by the less elongate male antennomeres starting from antennomere 3, the shorter female antennae (in *N.punctulifer*, female antennae distinctly surpass apex of elytra), and the presence of the rough longitudinal folds on male scape at least dorsally. Compared with *N.brudermanni* Holzschuh, 2020, the new species differs by the well-expressed median groove and the clearer and longer transverse folds on the pronotal disc, the male antennomere 3 being more strongly inflated in the apical part, and the male antennomere 4 being noticeably expanded apically. The new species also resembles *N.vitalisi* Pic, 1923, but differs by the sculpture of the pronotal disc, the longer median groove between upper eye lobes clearly extending onto the occiput, and the less elongate male antennomeres 3 and 4 (at least).

The new species should be attributed to the *unicolor* group sensu Miroshnikov (2020b), which includes *N.miaobenfui*.

#### Description.

Body length 69.0–77.0 mm, humeral width 20.0–22.0 mm. Body black to black-brown, partly reddish brown. Head with yellow-brown pubescence. Basal five antennomeres black-brown, covered with yellow-green to green-brown pubescence, remaining antennomeres more or less reddish brown, with a thinner pubescence, without a fringe of setae underneath.

Male antennae exceed apex of elytra by antennomere 8; scape stout, with a strongly protruding external apical angle, with well-expressed longitudinal folds dorsally, distinctly shorter than third antennomere, second antennomere strongly transverse, third to fifth antennomeres distinctly stouter than following, third very distinctly inflated in apical part, with an irregular rugose sculpture in basal half, fourth noticeably expanded apically, third distinctly longer than fourth; fifth distinctly more slender and longer than fourth, but shorter and stouter than sixth; sixth to eighth antennomeres subequal in length; ninth to eleventh gradually more slender and flatter apically, ninth slightly shorter than eighth and slightly longer than tenth; eleventh antennomere is the longest, as long as ninth and tenth combined. Female antennae distinctly fail to reach the apex of elytra; scape stout, with a strongly protruding external apical angle, approximately as in male, significantly shorter than third antennomere and slightly shorter than fourth; second antennomere very strongly transverse, third to fifth antennomeres slightly expanded in apical part, third subequal to fifth in length; fifth distinctly longer than fourth; sixth and seventh antennomeres more slender and semi-cylindrical, sixth distinctly longer than fifth, but distinctly shorter than fourth and fifth combined, seventh subequal to sixth in length, more slender than sixth; eighth to eleventh antennomeres become gradually flatter, eighth shorter than seventh, ninth shorter than eighth, tenth slightly shorter than ninth, eleventh longer than tenth.

Eye deeply emarginate, lower lobe very large; head with a deep longitudinal groove behind eyes on occiput. Mandible moderately sized, curved and sharp apically. Prothorax covered with dense yellow brown pubescence. Pronotum 1.29 or 1.20× as wide as long in male and female, respectively; at base distinctly wider than at apex; usually with an abrupt constriction at apex and a moderate constriction at base; with ~5 largely transverse folds, a pair of oblique longitudinal grooves and one medial glabrous line.

Scutellum covered with sparse pubescence excepting the medial glabrous line, widely rounded at apex. Elytra completely covered with uniform green-brown pubescence; moderately elongated, 2.50–2.60× as long as humeral width; approximately parallel-sided starting from base, rounded at apex. Venter with most sclerites pubescent. Prosternum with several transverse grooves before middle, with a glabrous groove before apex; prosternal process expanded posteriorly, with a pair of distinct apical tubercles; mesoventral intercoxal process emarginate apically, forming two lobes, with pubescence being denser on sides than on middle part; between coxae considerably wider than prosternal process; meso- and metasterna and abdominal sternites with fine dense punctation; metasternum with a very sharp median groove. Legs black-brown to reddish brown; moderately long; femora quite robust, tibiae slender and non-grooved; metatarsomere 1 slightly shorter than tarsomeres 2 and 3 combined. Last visible abdominal sternite at apex widely rounded, last visible abdominal tergite emarginate, forming two lobes apically, particularly distinct in females.

#### Etymology.

The new species is dedicated to Mr Mao-Bin Gu (顾茂彬), an entomologist from the Chinese Academy of Forestry, who deposited many longicorn beetles he collected in Hainan Island in IZCAS, including three of the type specimens of this new species.

#### Distribution.

China: Hainan.

### ﻿ Neocerambyx melas

Taxon classificationAnimaliaColeopteraCerambycidae

(Holzschuh, 2021)

27860F78-2820-5042-B3F0-677733C842AA

Fig. 6A–C

Massicus melas Holzschuh, 2021: 97, fig. 5. Type locality: Vietnam, Lao Cai Province, Sapa, Ta Phin, 1460 m, 22°23.37'N, 103°49.11'E.
Neocerambyx melas : Miroshnikov, 2022: 52.

#### Material examined.

China • 1♀; Fujian, Wuyishan, Sangang [福建武夷山三港]; alt. 740 m; 1 August 1997; Jian Yao leg.; IZCAS • 1♂; Fujian, Wuyishan, Guwankeng [福建武夷山古玩坑]; 8 July 2024; Chen-Qun Wu leg.; CLYQ.

**Figure 6.**
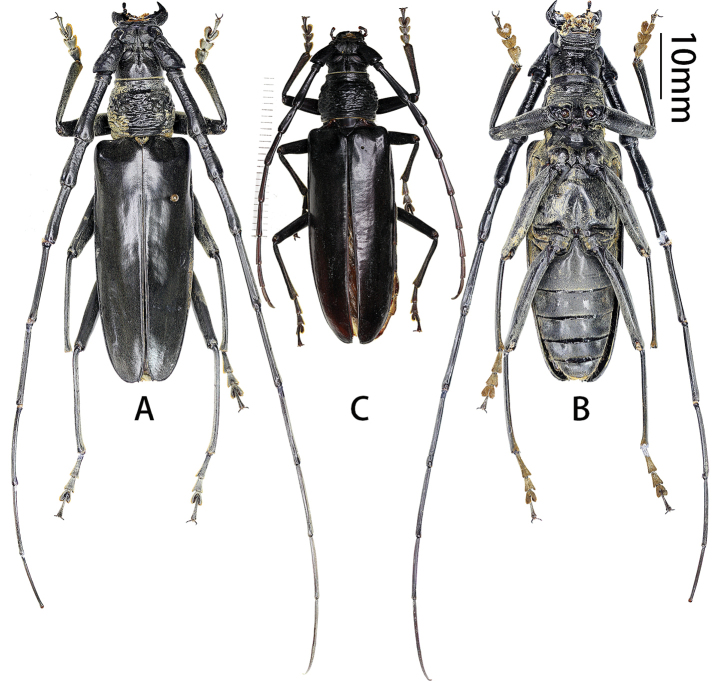
Habitus of *Neocerambyxmelas* (Holzschuh, 2021). **A, B.** ♂, from Fujian; **C.** ♀, from Fujian: **A, C.** Dorsal views; **B.** Ventral view.

#### Distribution.

Until now, this species has only been known from Northern Vietnam (Holzschuh 2021). In the material listed above, *Neocerambyxmelas* is here recorded from China (Fujian) for the first time. This species should be attributed to the *pellitus* group sensu Miroshnikov (2020b).

## Supplementary Material

XML Treatment for Neocerambyx

XML Treatment for Neocerambyx liyuani

XML Treatment for Neocerambyx miaobenfui

XML Treatment for Neocerambyx gui

XML Treatment for Neocerambyx melas

## ﻿Additional information

### Use of AI

No use of AI was reported.

### Author ORCIDs

Mei-Ying Lin https://orcid.org/0000-0001-9219-724X

Alexandr Miroshnikov https://orcid.org/0009-0000-5777-0442

Li He https://orcid.org/0000-0002-4597-3442

### Data availability

All of the data that support the findings of this study are available in the main text.
